# Identification of Transgene-Free CRISPR-Edited Plants of Rice, Tomato, and *Arabidopsis* by Monitoring DsRED Fluorescence in Dry Seeds

**DOI:** 10.3389/fpls.2019.01150

**Published:** 2019-09-18

**Authors:** Norma Aliaga-Franco, Cunjin Zhang, Silvia Presa, Anjil K. Srivastava, Antonio Granell, David Alabadí, Ari Sadanandom, Miguel A. Blázquez, Eugenio G. Minguet

**Affiliations:** ^1^Instituto de Biología Molecular y Celular de Plantas, Consejo Superior de Investigaciones Científicas (CSIC)—Universidad Politécnica de Valencia, Valencia, Spain; ^2^Department of Biosciences, Durham University, Durham, United Kingdom

**Keywords:** CRISPR/Cas9, genome editing, DsRED, *Solanum lycopersicum*, *Oryza sativa*, *Arabidopsis thaliana*

## Abstract

Efficient elimination of the editing machinery remains a challenge in plant biotechnology after genome editing to minimize the probability of off-target mutations, but it is also important to deliver end users with edited plants free of foreign DNA. Using the modular cloning system Golden Braid, we have included a fluorescence-dependent transgene monitoring module to the genome-editing tool box. We have tested this approach in *Solanum lycopersicum*, *Oryza sativa*, and *Arabidopsis thaliana*. We demonstrate that DsRED fluorescence visualization works efficiently in dry seeds as marker for the detection of the transgene in the three species allowing an efficient method for selecting transgene-free dry seeds. In the first generation of DsRED-free CRISPR/Cas9 null segregants, we detected gene editing of selected targets including homozygous mutants for the plant species tested. We demonstrate that this strategy allows rapid selection of transgene-free homozygous edited crop plants in a single generation after *in vitro* transformation.

## Introduction

CRISPR/Cas technology, adapted from bacterial immune system (Bolotin et al., 2005; Mojica et al., 2005), for specific and precise modification of genomes (Jinek et al., 2012; Wiedenheft et al., 2012) has transformed molecular biology. The efficiency of CRISPR/Cas9 genome-editing methodology was quickly demonstrated in plants (Feng et al., 2013; Jiang et al., 2013). *Arabidopsis thaliana* can be easily transformed *in planta* (Bechtold and Pelletier, 1998), but this is actually an exception because *in vitro* transformation is the most common methodology to generate stable transformed plants, which is a labor-intensive procedure that requires appropriate infrastructures, and more importantly, the regeneration process is slow and, depending on the species, it can range from several months to a year (Busov et al., 2005). Many markers have been used for selecting transformed plants, but the most commonly used are genes that confer resistance to antibiotics or herbicides (Miki and Mchugh, 2004). Markers are key elements for *in vitro* selection of cells with transgene integration, the regeneration of plants, and selection in the next generations; however, the presence of resistance genes raises concerns about biosecurity, and several strategies have also been developed for their elimination while retaining the genes of interest, or for using marker-free transformation strategies (Darbani et al., 2007; Yau and Stewart, 2013), but these strategies are neither shorter nor simpler. Thus, quick elimination of the transgene remains a challenge in plant biotechnology after genome editing, especially for crops due to their long life cycle and multiploidy, not only to avoid transgene position effects and to minimize the probability of off-target mutation appearance but also to deliver end users with edited plants free of the recombinant gene-editing machinery. Counter selection based on resistance marker genes are inconvenient in the case of CRISPR/Cas applications because plants lacking the transgene cannot survive the selection, and thus two more generations must be screened to evaluate the presence of the transgene. In the case of some crops, generations can last between 4 and 6 months and a few years, and the workload may be a limiting factor because transgene detection by PCR requires germination of seeds, so selected plants must be grown until the next generation can be harvested. The expression of fluorescent proteins as selective markers has been successfully used in *A. thaliana* (Stuitje et al., 2003; Shimada et al., 2010) as a fast method for transgene presence detection prior to seed germination. Moreover, it has also been used in combination with CRISPR in *Arabidopsis* (Gao et al., 2016; Durr et al., 2018; Wu et al., 2018; Yu and Zhao, 2019). Despite its clear advantage, it has not been tested in species such as tomato or rice, because of the special requirements of *in vitro* transformation protocols. To overcome the abovementioned technical difficulty, we have adapted fluorescence-mediated monitoring of transgenes to genome-editing approaches in these species with the goal of obtaining transgene-free homozygous gene edited crop plants in two generations.

## Materials and Methods

### Plant Material and Growth Conditions

Seeds of *A. thaliana* (ecotype *Col-0*) was stratified in agarose 0.05% (in water) for 3 days at 4°C, sown on pots containing soil mix (1:1:1 perlite, vermiculite, and peat [DSM]) and grown in greenhouse growth chambers at 22°C under long day conditions (16 h of light and 8 h of darkness). Rice (*Oryza sativa* L. cv. Nipponbare) seeds were put on ½ MS basal salts with 1% sucrose left in dark at 28°C for 2 days and then transfer to Sanyo growth chamber with light on for 1 week. The seedlings were planted in pots (8 × 8 × 10 cm) containing 180-g water-soaked soil. Plants were grown in white fluorescent light (600 photons m−2 s−1, 14 h of light/10 h of dark) at 28°C/25°C) and 70% relative humidity. Tomato (*Solanum lycopersicum* cv. money maker) seeds were sown on pots containing soil mix [1:2 perlite and peat (OPM)] and grown in greenhouse chambers at 24°C under long day conditions (16 h of light and 8 h of darkness). Each new generation was obtained leaving each plant to be autopollinated.

### Phylogenetic Analysis

BLAST against tomato (SGN) (Fernandez-Pozo et al., 2015) was performed using protein sequence of AtIAMT1 (At5g55250). The best four hits were used for alignment against IAMT1 protein sequences from *A. thaliana*, *Brassica rapa*, *Medicago truncatula*, and *O. sativa* using ClustalX2 (Larkin et al., 2007) with default parameters.

### sgRNA Target Selection

ARES-GT software (https://github.com/eugomin/ARES-GT.git) was used for identification and selection of CRISPR targets. Parameters for identification of possible off-targets were: less than five mismatches (L0 = 4) or less than four mismatches (L1 = 3) if one mismatch is found in seed sequence. Targets with no expected off-targets and close to the start of the ORF were selected (**Supplementary Table S1**).

### Plasmid Construction

**Figure 1:**
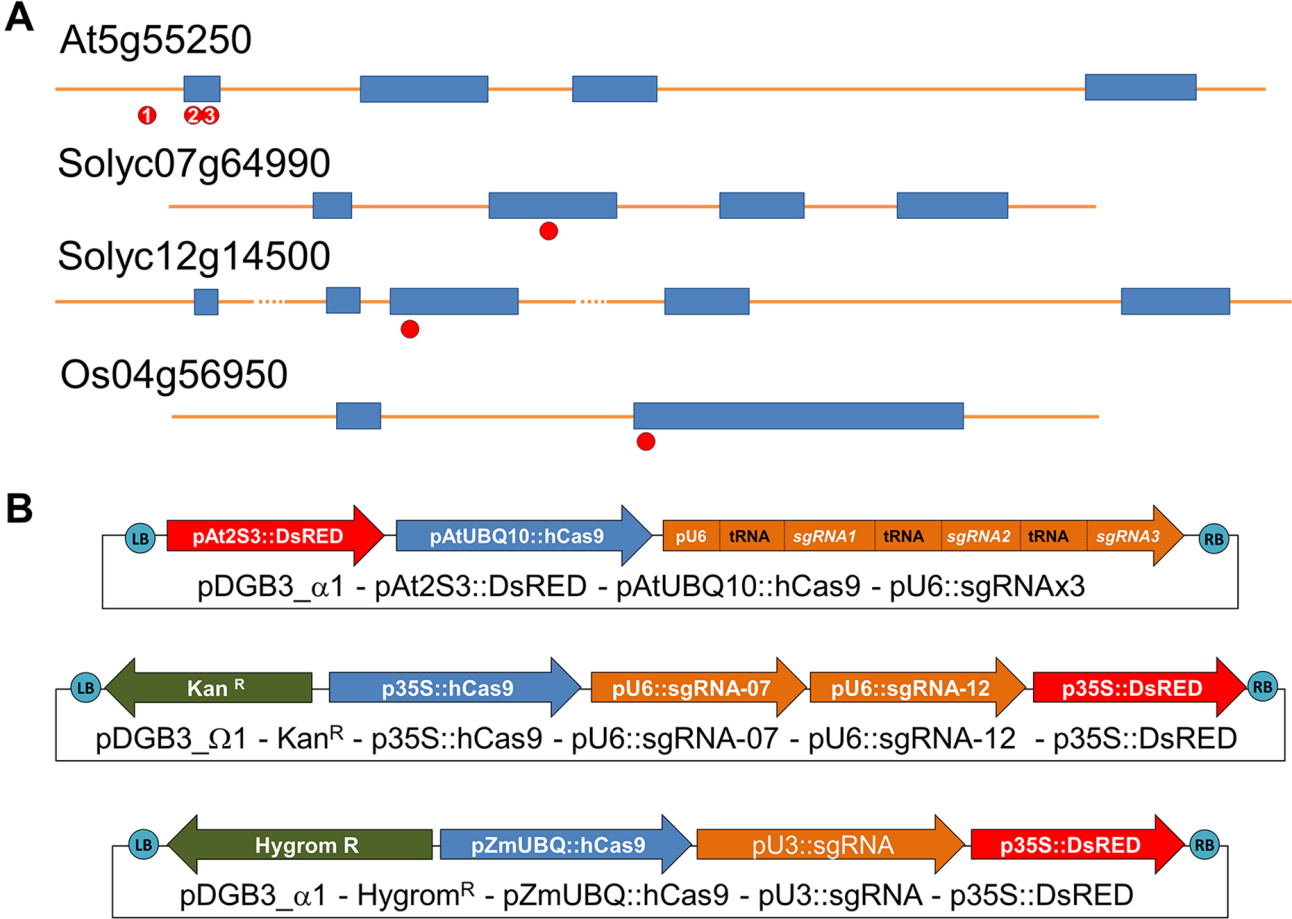
**(A)** Position of each CRISPR target in selected genes *At5g55250* (*Arabidopsis thaliana*), *Solyc07g64990* and *Solyc12g14500* (*Solanum lycopersicon*), and *Os04g56950* (*Oryza sativa*). **(B)** Description of transcriptional units (arrows) assembled in the vectors generated for plant transformation using the modular system GoldenBraid. Specific promoters were selected for expression of Cas9 protein in each species, pAtUBQ10 in *Arabidopsis thaliana*, p35S for *Solanum Lycopersicum*, and pZmUBQ for *Oryza sativa*. Adequate promoter for expression of sgRNA was selected: pAtU6-26 for dicotyledonous species (*Solanum* and *Arabidopsis*) and pOsU3 for monocotyledonous species (rice).

All vectors used in this work have been designed using GoldenBraid system following the described assembly strategy (Sarrion-Perdigones et al., 2013; Sarrion-Perdigones et al., 2014; Vazquez-Vilar et al., 2015; Vazquez-Vilar et al., 2016). We generated vectors containing the hCas9 CDS (GB0575) under the control of optimal promoters for each species: AtUBQ10 (GB2478), ZmUBQ (EGM001), and CaMV 35S (GB0030) for *Arabidopsis*, rice, and tomato, respectively. Similarly, the sgRNA multiplexed transcriptional unit (TU) were placed under the control of the AtU6-26 promoter (GB1001) for *Arabidopsis* and tomato, and the OsU3 promoter (GB1184) for rice (Vazquez-Vilar et al., 2016). Specific resistance genes were also introduced into each vector for *in vitro* selection, as required by crop transformation protocols: hygromycin (GB0211) and kanamycin (GB0226) for rice and tomato, respectively. Finally, an additional TU for expression of the fluorescent protein DsRED under the control of the At2S3 promoter (Kroj et al., 2003; Ravi et al., 2014; Bernabé-Orts et al., 2019) (GB2482) for *Arabidopsis* or CaMV 35S promoter (GB0361) for rice, and tomato was added in each final vector (**Figure 1B**; **Supplementary Figures 2** and **3**; **Supplementary Tables 2** and **3**). In all cases, TU used for primary transformant selection was placed at the LB. In the case of rice and tomato, TU for expression of DsRED was placed in the RB. Sequences of GB-Parts are accessible at GB cloning website (https://gbcloning.upv.es/) using the GB database ID.

### Plant Transformation

*Arabidopsis* was transformed by floral dip (Clough and Bent, 1998), with a minor modification: 1 min dipping into a solution (sucrose 5% + 0.2 ml Silwet-77/L) containing *Agrobacterium tumefaciens*. Protocol from Ellul et al. (2003) was followed for *in vitro* tomato transformation. All *in vitro* steps were carried out in a long-day growth chamber (16 h light/8 h dark, 24°C, 60–70% humidity, 250 μmol/m^2^/s). Protocol from Hiei and Komari (2008) till step 19 was used for *in vitro* transformation of rice; the regeneration (R-III) and rooting (HF) were done as described in Toki et al. (2006). Fluorescent seeds, containing the transgene, were identified in a Leica DMS1000 microscope with DsRED filter.

### Plant Genotyping

Genomic DNA was obtained from young leaves in all cases. CTAB extraction protocol (Murray and Thompson, 1980) was used in the case of tomato and rice, with small modifications: 600 μl of CTAB fresh buffer (2% CTAB, 1.4 M NaCl, 20 mM EDTA, 100 mM Tris-HCl pH 8.0, 2% β-mercaptoethanol) is added to 100 mg of ground tissue powder prior 45-min incubation at 65°C; then, 600 μl of chloroform:isoamyl alcohol (24:1) is added, extract is emulsified by vortex, and water phase is recovered after 10 min of centrifugation (13,000 rpm); then, 1 volume of 2-propanol is added and mixed gently and, after 20 min at 80ºC, centrifuged for 10 min at 4°C (13,000 rpm); pellet is washed once with ethanol, and after 5-min centrifugation (13,000 rpm), supernatant is discarded; finally, dry pellet is resuspended with 100 μl of Milli-Q water. In the case of *Arabidopsis*, the protocol described in Edwards et al. (1991) was used. PCR was performed using the specific primers for each fragment (**Supplementary Table S4**) and the MyTaq Red DNA Polymerase (BIO21110 Bioline-Ecogen) following manufacturer specifications. Macherey-Nagel NucleoSpin^®^ Gel and PCR Clean-Up Kit (ref. 740609.250) was used for PCR product purification, and sequences were determined by Sanger sequencing (GenoScreen services). Chromatograms of heterozygous and biallelics was analyzed by TIDE (Brinkman et al., 2014) and/or by visual inspection to determine the exact sequence of each allele.

## Results and Discussion

As a proof of concept, we decided to use the gene encoding IAA methyl transferase (*IAMT*) as a gene-editing target in three plant model species (*A. thaliana, O. sativa, and S. lycopersicum*), given that loss of function results only in difficulty for hypocotyl reorientation after gravistimulation (Abbas et al., 2018b) and increased pollen tube growth rate (Abbas et al., 2018a), neither of which are traits that can bias our identification of mutations by direct observation unless specific tests are performed. In *A. thaliana* and *O. sativa*, only one gene per species has been identified that encodes an enzyme with IAMT activity (At5g55250 and Os04g56950, respectively) (Qin et al., 2005; Yang et al., 2008). However, in tomato, we have identified two orthologs of IAMT1 (Solyc07g64990 and Solyc12g14500) by phylogenetic analysis (**Supplementary Figure 1**). Therefore, we decided to test different editing strategies in each of the three selected species: targeting only one gene with one sgRNA (in rice), simultaneously targeting two genes with two sgRNAs (in tomato), and targeting different regions of a single gene (in *Arabidopsis*) to evaluate the efficiency of the vectors when looking for multiple mutations and larger deletions. Genomic DNA sequence for each selected gene in each species was analyzed with ARES-GT tool for the identification and selection of sgRNAs (**Figure 1A**; **Supplementary Table 1**).

**Figure 2:**
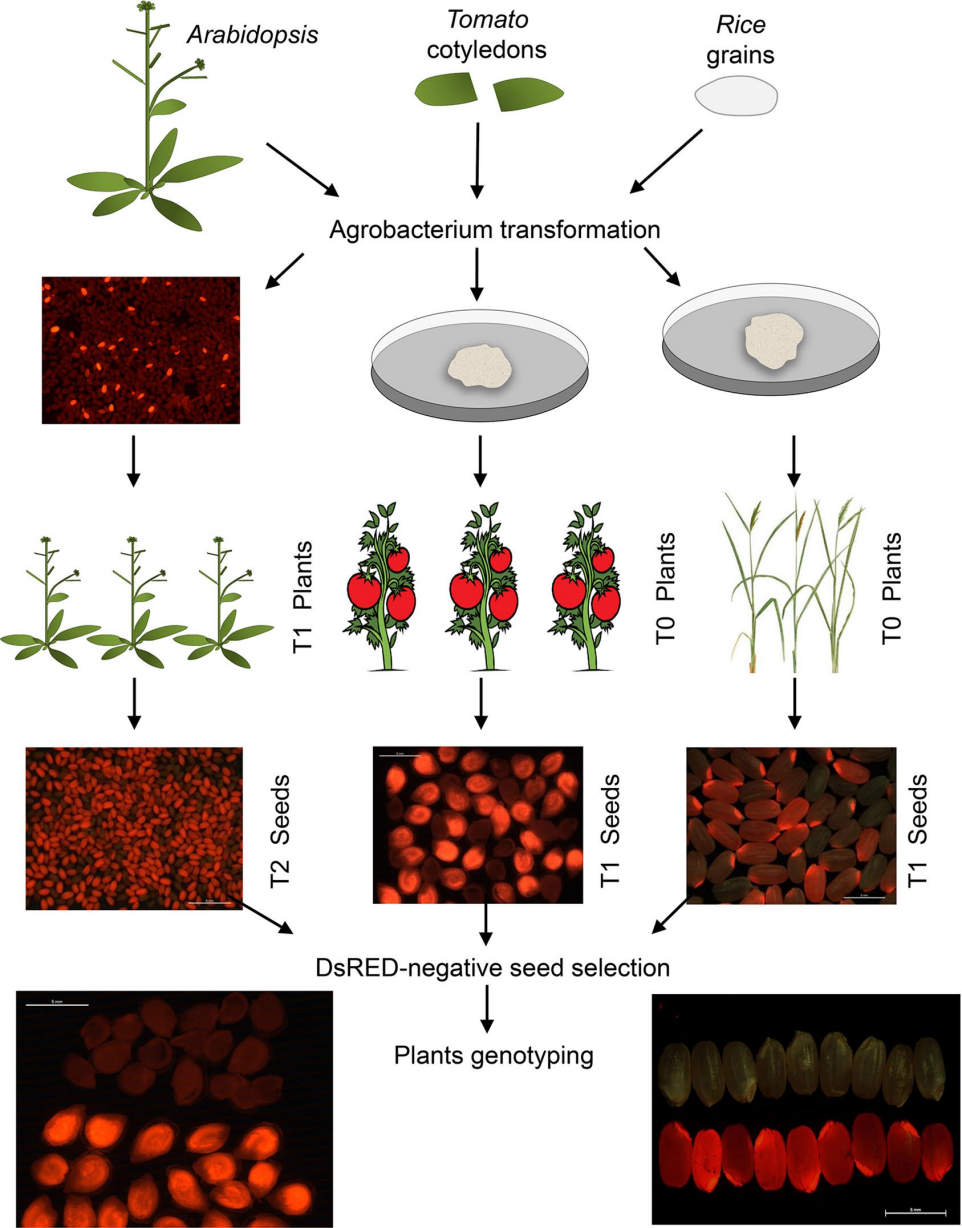
Diagram describing the steps for plant transformation and selection. Transformation of *Arabidopsis thaliana* was done by floral dip while *in vitro* transformation was used for *Solanum lycopersicum* and *Oryza sativa*. Selection of DsRED T1 seeds of *Arabidopsis thaliana* and DsRED-negative selection of segregant seeds from the three species was done by direct visualization under a stereoscope equipped with DsRED filter. Detection of fluorescence in seed is very clear, easy, and fast in all three species, and it allowed perfect separation of positive and negative fluorescent seeds.

Thanks to the modular design for construct generation through the GoldenBraid cloning system (Sarrion-Perdigones et al., 2013; Vazquez-Vilar et al., 2015; Vazquez-Vilar et al., 2016), we generated one vector for each plant species with all needed transcriptional units (**Figure 1B**; **Supplementary Figures 2** and **3**). Each final GoldenBraid construct was introduced into the corresponding plant species following published transformation protocols (see *Materials and Methods*) (**Figure 2**). Seeds from transformed *Arabidopsis* plants were harvested, and DsRED fluorescence was used to select 15 T1 seeds with high DsRED signal. *In vitro* selection of callus from tomato leaves and rice grains allowed the regeneration of eight kanamycin resistance transgenic tomato lines and 20 hygromycin resistance transgenic rice lines. Ideally, those primary transformant plants contain one copy of the transgene that will be segregated in the next generation independently of any CRISPR-induced mutations in germline; thus, we could use DsRED visualization as marker of transgene presence to select nonfluorescent seeds and then search for mutations. While all primary transformants of rice and *Arabidopsis* produced seeds, two of the tomato plants presented a dwarf phenotype and did not produce any fruit. Segregation analysis was done by visual observation of segregant dry seeds under a stereoscope equipped with DsRED filter. While signal in tomato presented a homogeneous pattern in the embryo in all lines, in rice, the intensity of DsRED signal in embryo and endosperm varies between lines (**Supplementary Figure 4**). First, we discarded two rice lines and two tomato lines in which no DsRED seeds were observed. Based on the expected 3:1 ratio of DsRED fluorescent *vs.* non-fluorescent seeds for one T-DNA insertion, 12 *Arabidopsis*, 4 tomato, and 14 rice lines were retained for further analysis (**Table 1**). It is worth noting that with DsRED-negative selection of segregants, lines with multiple insertions are not necessarily unwelcome. Those lines are usually discarded because stablishing stable transgenic lines need more work and very careful analysis of segregation of the different insertions. However, the use of DsRED counterselection facilitates the identification of non-transgenic seeds despite the very low frequency in which they may be present as a result of multiple insertions (for example, 1:15 in the case of two insertions). This is an advantage for species with low transformation rate or to maximize CRISPR efficiency. Of course, as in any *Agrobacterium*-mediated transformation method, incomplete transgene copies can be incorporated in some occasions thus absence of any truncated copy should be confirmed in selected mutated lines.

**Table 1:** Number of independent transgenic lines used for each species and genotyping of transgene-free T2 Plants.

Species	Target	Number of Primary transformants	Number of independent lines (3:1 ratio)	Number of selected lines	Number of T2/T1 plants	Genotype T2/T1 plants				
						Mut. Hom.	Biallelic	Heter.	wt	WT*
Oryza sativa	Os04g56950	20	14	14	31	20 (65%)	8 (26%)	2 ( 6%)	1 (3%)	1 (3%)
Solanum lycopersicum	Solyc07g64990	8	4	4	29	2 (7%)	0	3 (10%)	24 (83%)	6 (21%)
	Solyc12g14500					11 (38%)	12 (41%)	0	6 (21%)	
Arabidopsis thaliana	At5g55250-1	15	12	4	214	0	0	3 (1.4%)	211 (98.6%)	112 (52%)
	At5g55250-2					9 (4.2%)	5 (2.3%)	14 (6.5%)	186 (86.9%)	
	At5g55250-3					42 (19.6%)	2 (0.9%)	43 (20.1%)	127 (59.3%)	

Each genomic region was PCR amplified and sequencing to determine genotype of each genomic target and classify in four groups: both chromosomes mutated with the same mutation (Mut. Hom.); both chromosomes mutated but with different mutations (biallelic), only one chromosome with mutation (Heter.), and no detected mutation (wt). *The last column indicates de number of plants with wild-type sequence in all CRISPR targets.

**Figure 3:**
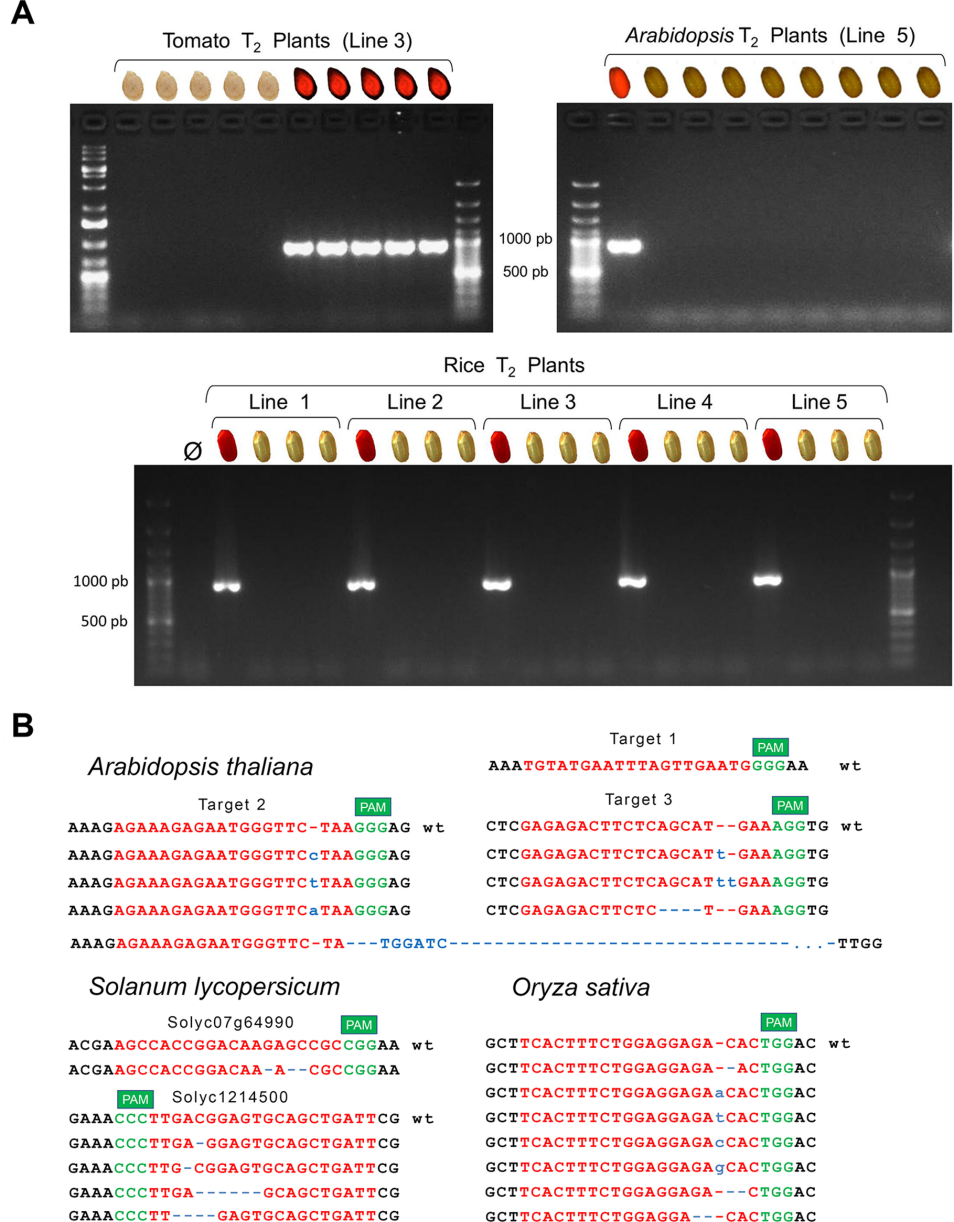
**(A)** Absence of transgene in plants from DsRED-negative selected seeds was confirmed by PCR using *Cas9*-specific primers. Examples are shown for tomato, *Arabidopsis* and rice, selecting different number of seeds and number of independent lines. In all cases, fluorescence and Cas9 PCR band did correlate perfectly (an image indicates the presence/absence of DsRED fluorescence in the original selected seed). **(B)** Sequence alignment of mutations detected in homozygosis in transgene-free T2 individual plants. CRISPR target sequence in red and PAM sequence in green. Nucleotide insertions are indicated in blue; deletions are also indicated with blue dashes. Deletion “del193” starts in target 2 and ends around 150 nts downstream target 3. It also includes an insertion of six nts that interestingly match with six nucleotides upstream target 2 (**Supplementary Figure 5B**).

First, we evaluated DsRED as effective transgene marker; thus, we selected seeds with and without DsRED signal from a few lines of each species to germinate and grow them under optimal greenhouse conditions until leaves were available for genomic DNA extraction. We confirmed that the Cas9-specific band was detected by PCR only in the plants from the DsRED-positive seeds (**Figure 3A**) in the three species. Next, we selected negative DsRED seeds because our main interest is the efficient identification of mutations in transgene-free plants; then, each CRISPR-target genomic region was PCR-amplified and sequenced only from individual plants originated from non-fluorescent seeds. We confirmed the presence of mutations in most of the transgene-free plants from all species (**Table 1**; **Figure 3B**; **Supplementary Tables 5–10**), indicating that stable mutations had been generated in the previous generation and inherited through the germline independently of transgene. Most of the plants presented mutations in heterozygosis, but more importantly, in all species, we did identify plants with mutations in homozygosis.

In the case of tomato, where two different genes were targeted at the same time, eight DsRED-negative seeds were selected from each selected line, and the T1-germinated plants on soil were analyzed. We identified four different deletions in gene *Solyc12g14500* in homozygosis in plants from three of the four lines; however, we only detected an atypical deletion of three nucleotides in homozygosis in *Solyc07g64990* in one of the lines (**Figure 3B**; **Supplementary Table 5**). Variability in CRISPR-Cas9 efficiency has been widely described suggesting that accessibility of DNA or sequence composition of target can affect efficiency (Campenhout et al., 2019), but we can not discard a deleterious effect of a null mutant in that gene neither, albeit is not an objective in this work to elucidate it. Only 21% of all T1 plants did not present any mutation (actually, it corresponds to the six plants from line 6, in which no mutations were detected), while all the other T1 plants presented mutations in gene *Solyc12g14500* either as biallelic or in homozygosis. Due to the low rate of mutations found in Solyc07g64990 target, most of the plants were wild type for Solyc07g64990 and mutated for Solyc12g14500. Actually, only two plants had both genes edited in homozygosis.

In rice, with 14 independent lines, 3 T1-negative DsRED seeds of each line were selected to be sown on soil. All germinated plants were analyzed, and mutations in target position were detected in plants from all lines (**Supplementary Table 6**). Only one T1 plant (from line 18) presented the wild-type allele in homozygosis, and we also found it in heterozygosis in two additional T1 plants from line 20. In line 4, the three T1 plants did present the same mutation in homozygosis (insertion of one “A”), suggesting that the corresponding T0 plant likely was already homozygous. We selected five new DsRED-negative seeds from that line, and we confirmed that all plants presented the same “A” insertion in homozygosis. The absence of wild-type allele was also observed in other lines, in which only mutated alleles were detected, both in heterozygosis (biallelic) and homozygosis, suggesting that both copies of the gene were mutated in the corresponding T0 plant. Taken into account all rice T1 plants, most of the plants had a mutation in homozygosis from which we identified three different deletion alleles and the four possible insertions of one nucleotide (**Figure 3B**; **Supplementary Table 6**).

In the case of *Arabidopsis*, we decided to select only four lines but a higher number of T2 plants to evaluate the presence of large deletions, spanning the whole region between two target sites. Between 50 and 80 DsRED-negative seeds were selected and sown on soil, and the germinated T2 plants were analyzed. Different individual mutations in homozygosis in two of the three targets were detected (**Figure 3B**; **Supplementary Tables 7–10**), while we only detect one plant with a small deletion (3 pb) in heterozygosis affecting target 1 (**Supplementary Table 10**). As the three targets are expressed in the same transcript using the strategy of multiplexing with tRNA autoprocessing (Xie et al., 2015), the result indicates a very low efficiency of RNA guide 1. Although the three targets matched the same genomic region, *Arabidopsis* presented the higher percentage of nonmutated T2 plants in comparison with rice and tomato. A deletion of 193pb affecting targets 2 and 3 was detected in homozygosis in three T2 plants from line 5, in addition to 2 heterozygote plants with the same deletion. A different big deletion (240 pb) was also detected in two plants of line 3 affecting targets 1 and 2, but only in heterozygotes (**Supplementary Figure 5A** and **Supplementary Table 10**). Visual inspection of chromatograms was allowed to determine the exact sequence of both deletions (**Supplementary Figure 5B**). Deletion del193 contains an insertion of six nucleotides that interestingly match six nucleotides upstream target 2. Insertions inside deletions have been previously described in genome editing (Bernabé-Orts et al., 2019), and it can be a result of DNA repairing mechanisms as MMEJ (Mcvey and Lee, 2008).

Taken together, these results are consistent with variable efficiency of sgRNAs reported in the literature, thus confirming that our vectors work as efficiently as other vector systems but with the advantage of using DsRED fluorescence as marker for transgene presence in dry seeds. It is noteworthy to mention that DsRED fluorescence has not decayed in seeds of the three species stored for almost 2 years. The identification of homozygous mutant plants has been successful in all cases despite the use of a low number of transformants. However, the same alleles are shared by all plants derived from the same individual primary transformant, thus exploring more independent lines is expected to be more effective in generating multiple alleles than increasing the number of selected individuals. From our data, the selection of only three seeds from each rice line instead of reducing the number of lines and selecting more T1 seeds did not reduce our efficiency in the number of different alleles found in homozygosis in comparison with tomato. Actually, the same mutations would be identified if only the first three plants of each tomato line are used compared with result from all tomato plants; only deletion of one “C” in homozygosis is missing though it would be detected in biallelic plants. Thus, this strategy could be advisable when the number of plants that can be growth is limited.

We have demonstrated that the use of DsRED fluorescence as a selectable marker of transgene in dry seeds is a robust method that facilitates the identification of transgene-free CRISPR-/Cas9-edited plants of rice and tomato in the second generation, minimizing the probability of off-target mutations. Seed DsRED fluorescence selection works in *A. thaliana* related species as *Camelina sativa* (Morineau et al., 2017) or *Cardamine hirsuta* (unpublished own data). Due to possible interference because of autofluorescence in plant tissues, different fluorescent protein must be evaluated to adapt vectors to different species with agronomical interest. We have shown that DsRED is a very good option for rice and tomato and probably for many species, as shown by a recent report with wheat (Okada et al., 2019). A limitation in many vector systems is the laborious task of adapting them to new species, changing promoters and transcriptional units. GoldenBraid cloning system ensures easy modification of vectors for other Cas proteins, like Cas12a/Cpf1 (Bernabé-Orts et al., 2019), other fluorescence proteins, and required resistance genes, meaning that generation of new vectors for *in vitro* transformation is not a limiting factor. This approach can easily be extended to additional crops and model plants, as long as optimal promoters are available.

## Data Availability

All datasets for this study are included in the manuscript and the **Supplementary Files**.

## Author Contributions

EM, MB and DA contributed conception and design of the study; NA-F and EM designed and generated the Golden Braid vectors and transformed Arabidopsis thaliana; SP and AG transformed tomato; CZ, AKS and AS transformed rice; EM and NA-F analysed T2 lines; EM and MB wrote the first draft of the manuscript; AS, AG, DA, CZ and NA-F contributed to manuscript revision. All authors read and approved the submitted version.

## Funding

Grants AGL2014-57200-JIN (EGM), BFU2016-80621-P (MB), and BIO2016-78601-R (AG) from the current Spanish Ministry of Science, Innovation and Universities. Grants TRADITOM (634561), TomGEM (679796), Newcotiana (760331-2) and Pharma-Factory (SEP-210417525) from European Union H2020 program (AG). ERC grant “SUMOrice” and BBSRC grant “Flooding tolerance in rice” (AS).

## Conflict of Interest Statement

The authors declare that the research was conducted in the absence of any commercial or financial relationships that could be construed as a potential conflict of interest.
