# Supplementary material for: Identification of Transgene-Free CRISPR-Edited Plants of Rice, Tomato, and Arabidopsis by Monitoring DsRED Fluorescence in Dry Seeds
Source: Front Plant Sci. 2019 Sep 18;10:1150. doi: 10.3389/fpls.2019.01150 (PMC6759815; doi:10.3389/fpls.2019.01150)
Supplement: Supplementary file 1 [file DataSheet_1.pdf]

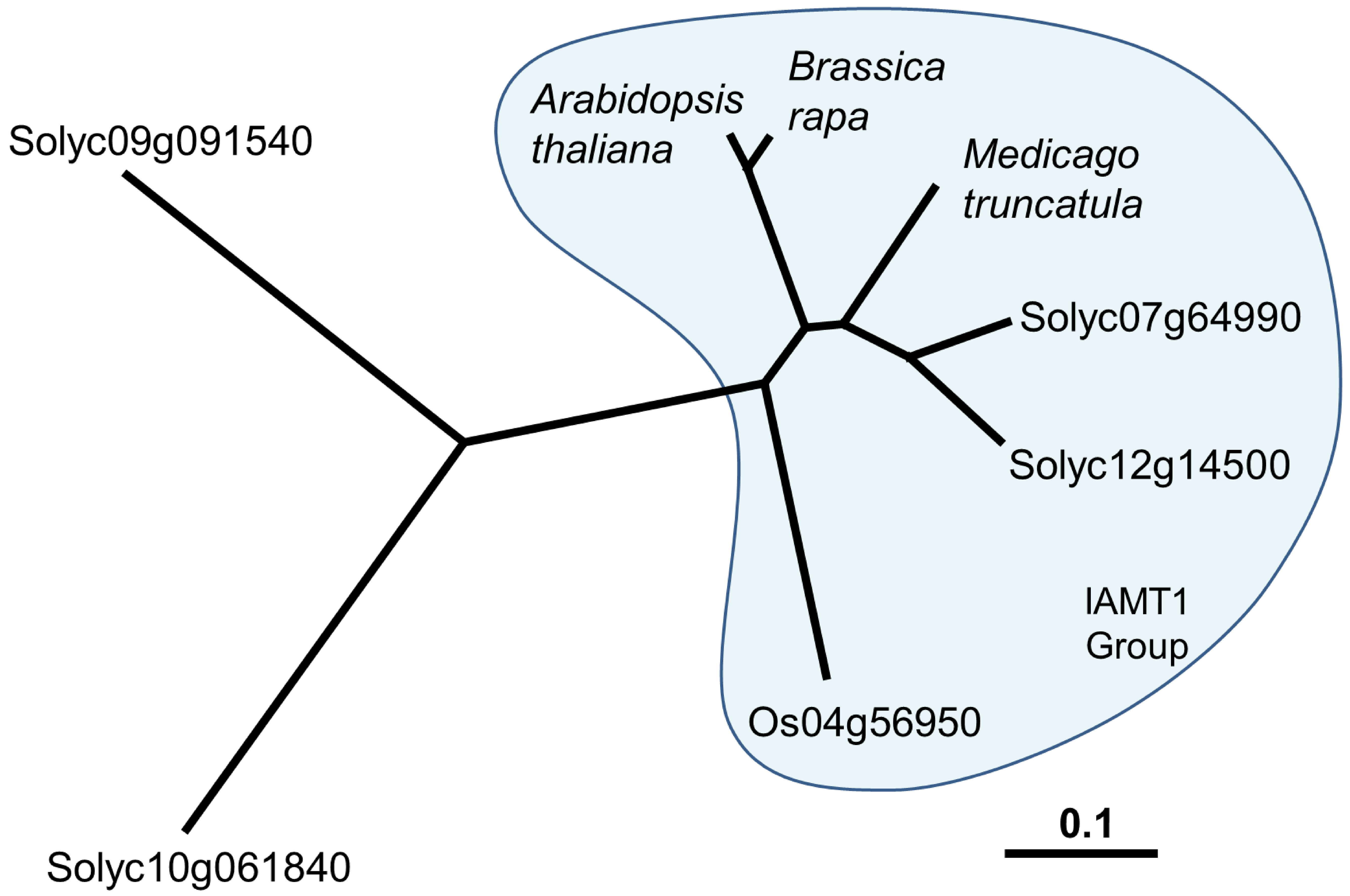

Supplemental Figure 1: Phylogenetic analysis for the identification ot the *IAMT1* orthologs candidates in Solanum Lycopersicum. Only genes *Solyc07g64990* and *Solyc12g14500* were grouped with the other plant IAMT1 (Brassica rapa: *LOC103844968*; Medicago truncatula: *Medtr3g054330*).

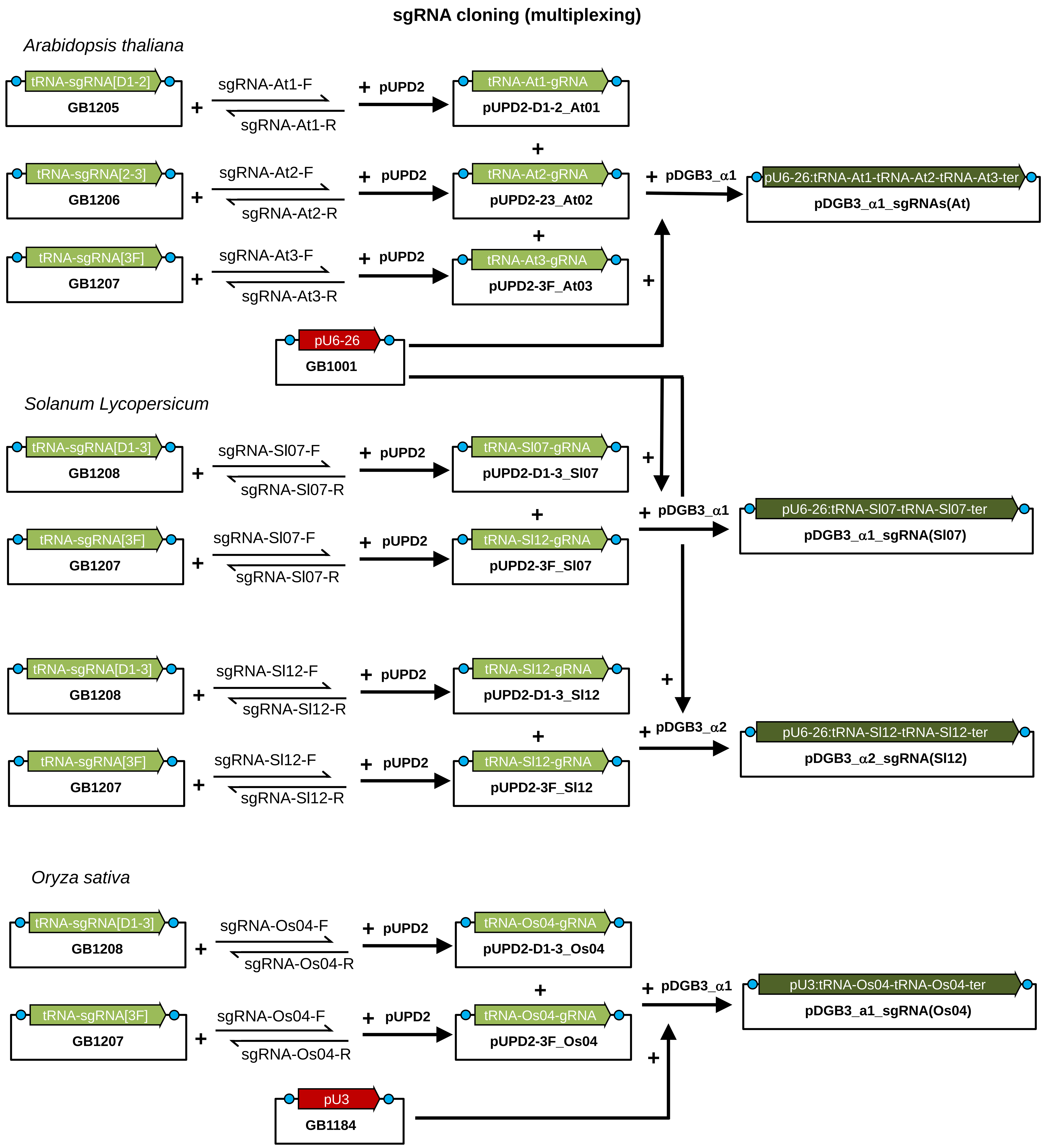

Supplemental Figure 2. Sequencial steps for sgRNA cloning (multiplexing) and generation of sgRNA transcriptional units in pDGB3 vectors.

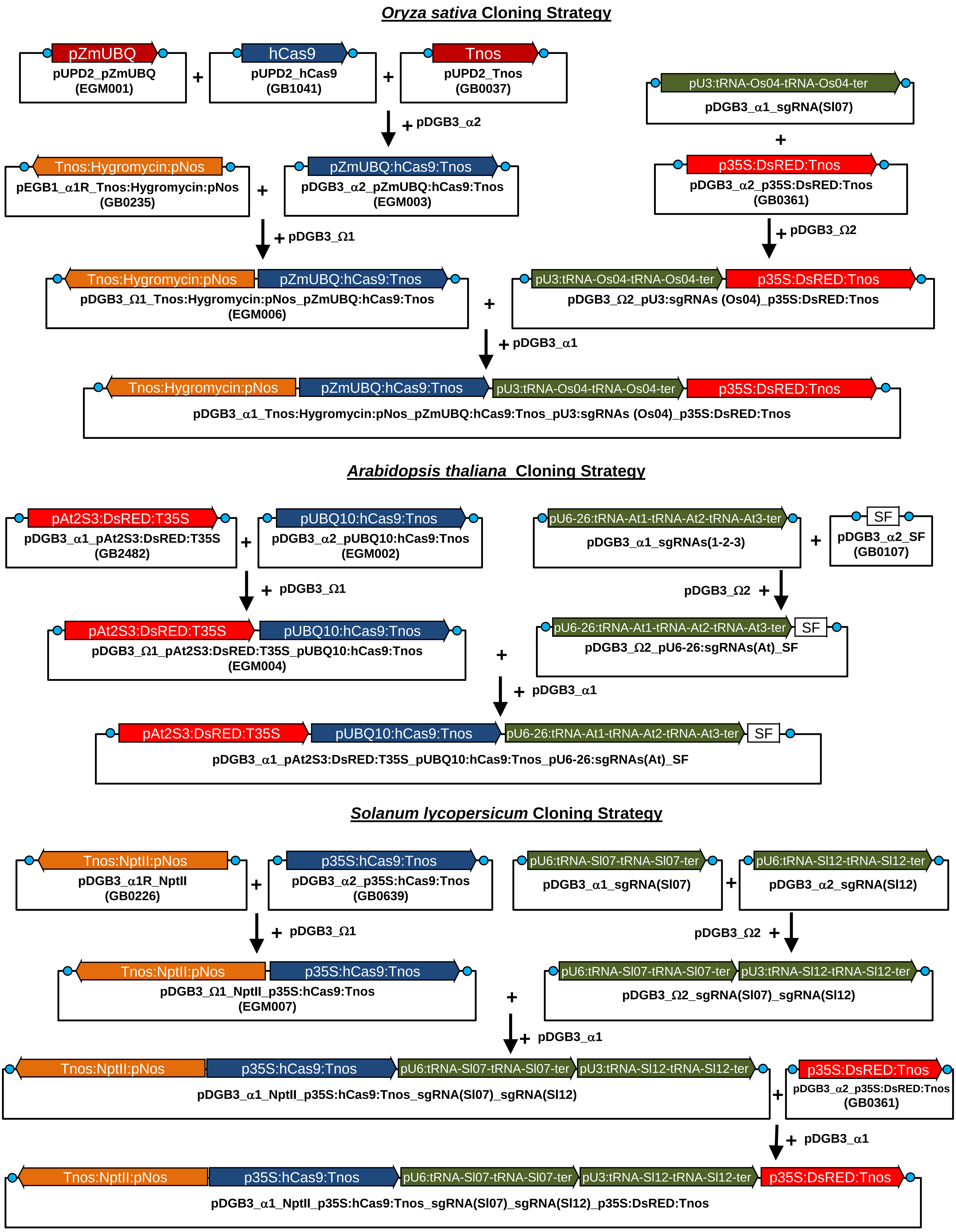

Supplemental Figure 3. Sequencial steps to assemble all transcriptional units needed for each final vector for plant transformation.

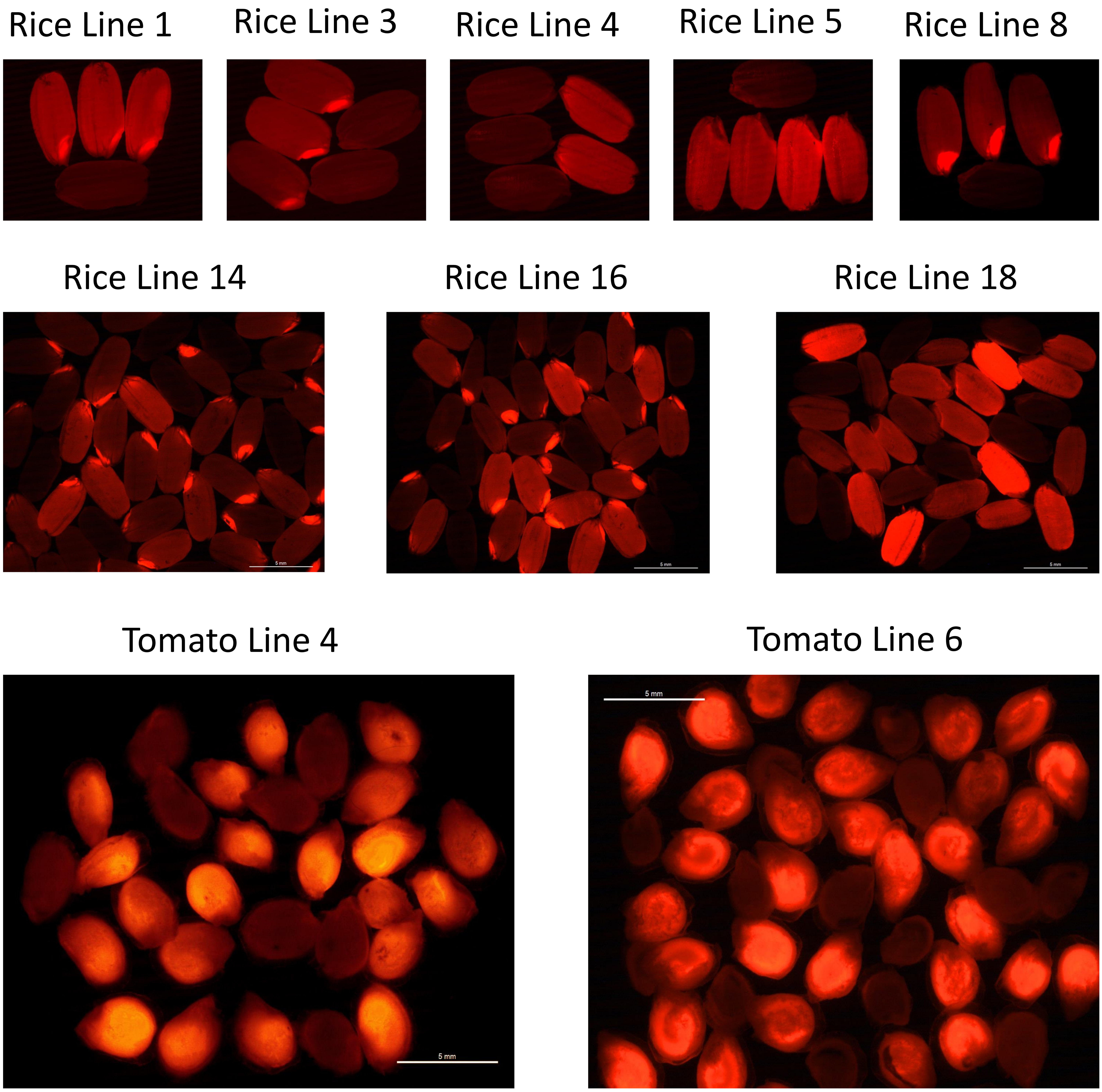

Supplemental Figure 4. DsRED signal in T2 segregants seeds from different independent lines of rice and tomato. We observed a similar embryo homogeneous pattern of DsRED signal in all tomato lines. In rice, DsRED signal can be observed in embryo and/or endosperm, and we have observed differences in the pattern of DsRED signal in different lines.

(A)

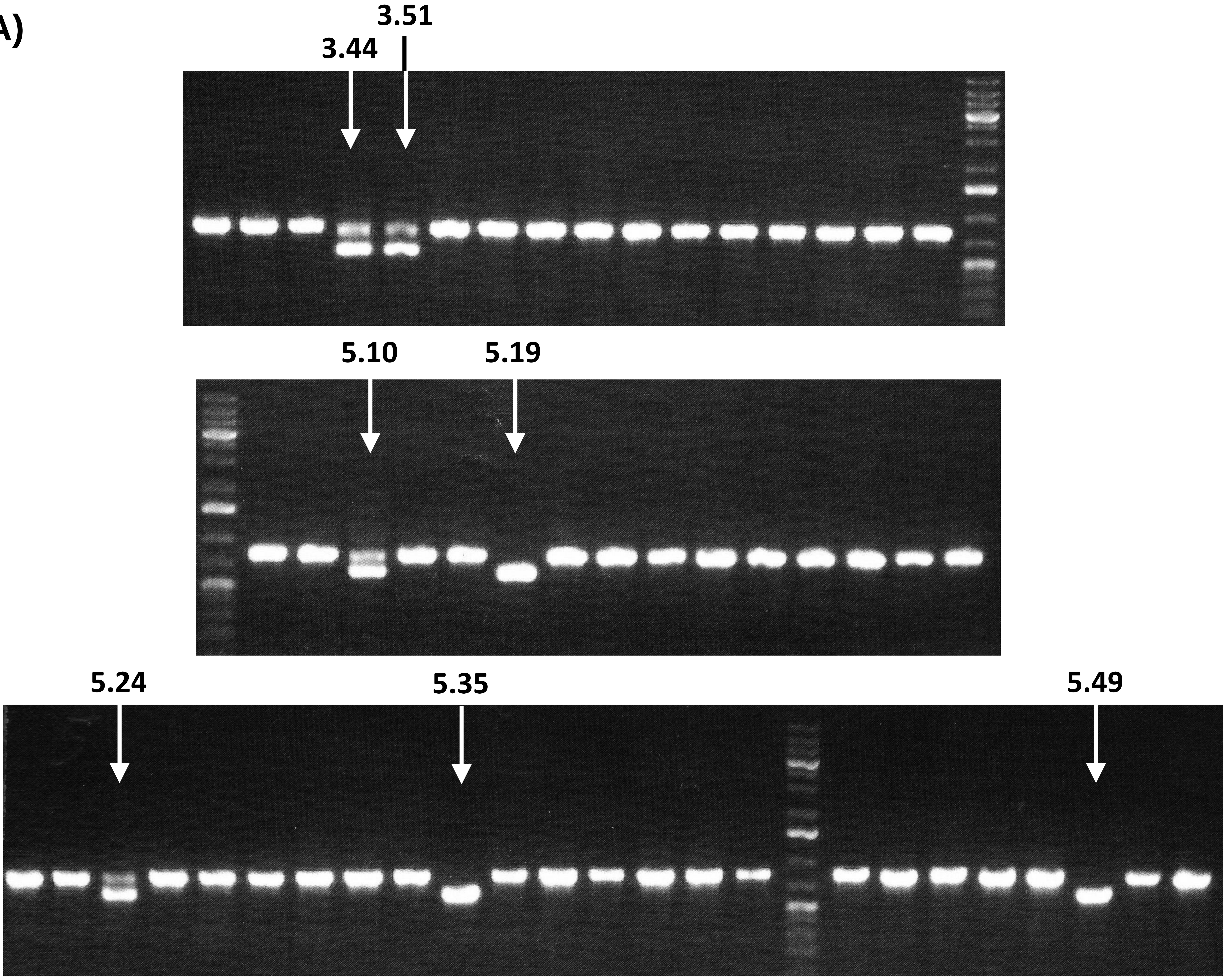

(B)

```
                                                                  Target 1
wt      TGTTTTTGCATGAATAATCAGTATATACAAAATATGTACGCAATGGCACATTTAATAATATGTTTCCCCATTCAACTAAATTCATACATTTAT
del193  TGTTTTTGCATGAATAATCAGTATATACAAAATATGTACGCAATGGCACATTTAATAATATGTTTCCCCATTCAACTAAATTCATACATTTAT
del240  TGTTTTTGCATG---------------------------------------------------------------------------------

wt      CATTTCATCTCTTTGTCCTCTTTTCCTATATATATGTGTCGTCTCCTCGAGCTTGTCTCATTCACAAAAAGAAGGTGTAGAGAAAGTTAAAGA
del193  CATTTCATCTCTTTGTCCTCTTTTCCTATATATATGTGTCGTCTCCTCGAGCTTGTCTCATTCACAAAAAGAAGGTGTAGAGAAAGTTAAAGA
del240  ---------------------------------------------------------------------------------------------
                                      Target 2                                         Target 3
wt      GAGAAGGAGAGATCCATAGAGAAAGAGAAAGAGAATGGGTTCTAAGGGAGACAACGTTGCTGTATGTAACATGAAGCTCGAGAGACTTCTCAG
del193  GAGAAGGAGAGATCCATAGAGAAAGAGAAAGAGAATGGGTTCTA----------TGGATC---------------------------------
del240  ------------------------------------------------------------------TAACATGAAGCTCGAGAGACTTCTCAG

wt      CATGAAAGGTGGCAAAGGACAAGACAGCTACGCCAATAACTCTCAAGCTCAGGTCTGTACATCTTATTTTTTTTTCTAATTACTTAAAAGTT
del193  --------------------------------------------------------------------------------------------
del240  CATGAAAGGTGGCAAAGGACAAGACAGCTACGCCAATAACTCTCAAGCTCAGGTCTGTACATCTTATTTTTTTTTCTAATTACTTAAAAGTT

wt      CCATTAAAAAGCCTTCTTATTATTCTATTTTCTATATCTCGAGTGATAAATTTGGGAATTTTAAGTGAAGGGACTGTGCTCAAGATTGGCAAG
del193  ---------------------------------------------------TTGGGAATTTTAAGTGAAGGGACTGTGCTCAAGATTGGCAAG
del240  CCATTAAAAAGCCTTCTTATTATTCTATTTTCTATATCTCGAGTGATAAATTTGGGAATTTTAAGTGAAGGGACTGTGCTCAAGATTGGCAAG
```

Supplemental Figure 5. A) Detection of big deletions in Arabidopsis. Agarose electrophoresis of PCR amplification of *IAMT1* sgRNA target genomic region from T2 plants of *Arabidopsis* lines 3 (del240) and 5 (del193). White arrows mark the lanes showing a lower size band, indicating the presence of a big deletion. In a few cases a doble band is observed, which indicates an heterozygous individual for the deletion. B) Sequence alignment of deletions "del193" and "del240". In red 5'UTR, in blue first exon, in light green first intron and deleted sequence is indicated with dark green dashes. In del193 6 nucleotides "TGGATC" (in orange) are inserted between deletion limits that did not corresponde with anything in deleted sequence though interestingly it corresponde with a sequence in the 5'UTR but in inverted orientation (orange line). CRISPR targets are indicated by red lines and PAM with green line.

Supplemental Table 1. Selected CRISPR target sequences.

Gene	Target	Sequence - PAM
At IAMT1 (At5g55250)	sgRNA-1	TGTATGAATTTAGTTGAATG GGG
At IAMT1 (At5g55250)	sgRNA-2	AGAAAGAGAATGGGTTCTAA GGG
At IAMT1 (At5g55250)	sgRNA-3	GAGAGACTTCTCAGCATGAA AGG
Solyc07g64990	sgRNA-07	AGCCACCGGACAAGAGCCGC CGG
Solyc12g14500	sgRNA-12	AATCAGCTGCACTCCGTCAA GGG
Os04g56950	sgRNA-04	TCACTTTCTGGAGGAGACAC TGG

Supplemental Table 2. GB parts generated in this study. Sequences are accessible at GB cloning website using the GB database ID.

**Gbparts generated in this study**		
**GB database ID**	**Name**	**Category**
EGM001	*Zea Mays* UBQ promoter	PROM+5'UTR
EGM002	pAtUBQ10::hCas9::Tnos	TU
EGM003	pZmUBQ::hCas9::Tnos	TU
EGM004	pAt2S3::DsRED::T35S<br>pAtUBQ10::hCas9::Tnos	MODULE
EGM005	pAt2S3::DsRED::T35S<br>pAtUBQ10::hCas9::Tnos_SF	MODULE
EGM006	Tnos:Hygromycin:pNos<br>pZmUBQ:hCAS9:Tnos	MODULE
EGM007	NptII(Kan^{R})_p35S:hCas9Tnos	MODULE

Supplemental Table 3. GB parts used in this study. Sequences are accessible at GB cloning website using the GB database ID.

**Gbparts used in this study**		
**GB database ID**	**Name**	**Category**
GB0030	*CaMV 35S*	PROM+5'UTR
GB0037	*Tnos*	3'UTR+TERM
GB1041	*hCas9*	CDS
GB1001	pAtU6-26 (PROM DPolIII)	PROM
GB1184	pOsU3 (PROM MPolIII)	PROM
GB2478	*A.thaliana UBQ10*	PROM+5UTR
GB1205	tRNA-gRNA position [D1_2]	Multiplexing (level -1)
GB1206	tRNA-gRNA position [2_3]	Multiplexing (level -1)
GB1207	tRNA-gRNA position [3F]	Multiplexing (level -1)
GB1208	tRNA-gRNA position [D1_3]	Multiplexing (level -1)
GB1209	tRNA-gRNA position [M1_2]	Multiplexing (level -1)
GB1210	tRNA-gRNA position [M1_3]	Multiplexing (level -1)
GB0107	pCambia_a2_SF	TU
GB0211	Hygromycin resitance	TU
GB0226	Kanamycin resistance (NptII)	TU
GB0361	*p35S::DsRED::Tnos*	TU
GB0639	*p35S::hCas9::Tnos*	TU
GB2482	*pAt2S3::DsRED::T35S*	TU

Supplemental Table 4. Primers used in this work.

Primer	Sequence
GB_PromZmUBIQ_for1	GCCGTCTCGCTCGGGAGGCATGCCTGCAGTGCAGCGT
GB_ZmUBIQ_rev1	GCCGTCTCGCTCACATTGTGAAGGGGGCGGCCGCGGA
sgRNA-At1-F	GTGCAGAGAGACTTCTCAGCATGAA
sgRNA-At1-R	AAACTTCATGCTGAGAAGTCTCTCT
sgRNA-At2-F	GTGCAAGAAAGAGAATGGGTTCTAA
sgRNA-At2-R	AAACTTAGAACCCATTCTCTTTCTT
sgRNA-At3-F	GTGCATGTATGAATTTAGTTGAATG
sgRNA-At3-R	AAACCATTCAACTAAATTCATACAT
sgRNA-Sl07-F	GTGCAAGCCACCGGACAAGAGCCGC
sgRNA-Sl07-R	AAACGCGGCTCTTGTCCGGTGGCTT
sgRNA-Sl12-F	GTGCAAATCAGCTGCACTCCGTCAA
sgRNA-Sl12-R	AAACTTGACGGAGTGCAGCTGATTT
sgRNA-Os04-F	GTGCATCACTTTCTGGAGGAGACAC
sgRNA-Os04-R	AAACGTGTCTCCTCCAGAAAGTGAT
gAtIAMT1-F	ACCTACAGTGCACAAAATGACA
gAtIAMT1-R	CTTGCCAATCTTGAGCACAGTC
gSl07-64990_F	TGGCACCTTTAGGAGACAATAATAACA
gSl07-64990_R	AACCAGGAACTCCGGCGGCGAAA
gSl12-14500_F	GCATGAAAGGAGGCAAAGGTGAAGC
gSl12-14500_R	CGCCGGGAATAGTCGCCGGTAAA
Os04g56950_F	GGATCTGAACTGAACCCTTGTGCT
Os04g56950_R	TTGCTGGGGAGGTCGGAGAA
g_hCas9_For	GGCGGAGCAAGCCAGGAGGAA
g_hCas9_Rev	CTTGACAGCCGCCCCCATCCT

Supplemental Table 5. Genotype and mutations of the tomato T2 plants used in this work.

Solanum Lycopersicum										
		**Solyc07g64990**				**Solyc12g14500**				
**Line**	**Plant**	**Genotype**			**Mutation**	**Genotype**			**Mutation**
**1**	1			wt		Mut. hom			del4
	2			wt			bi-allelic		del4/+1(T)
	3			wt		Mut. hom			del4
	4			wt		Mut. hom			del4
	5			wt			bi-allelic		del4/+1(T)
	6			wt			bi-allelic		del4/+1(T)
	7			wt			bi-allelic		del4/+1(T)
	8			wt			bi-allelic		del4/+1(T)
**3**	1		het		wt/del3		bi-allelic		-1(A)/-1(C)
	2		het		wt/del3		bi-allelic		-1(A)/-1(C)
	3			wt		Mut. hom			-1(A)
	4			wt		Mut. hom			-1(A)
	5		het		wt/del3		bi-allelic		-1(A)/-1(C)
	6			wt			bi-allelic		-1(A)/-1(C)
	7	Mut. Hom			del3	Mut. hom			-1(A)
	8	Mut. hom			del3	Mut. hom			-1(C)
**4**	1			wt		Mut. hom			-1(A)
	2			wt		Mut. hom			del6
	3			wt			bi-allelic		del6/-1(A)
	4			wt			bi-allelic		del6/-1(A)
	5			wt		Mut. hom			del6
	6			wt		Mut. hom			-1(A)
	7			wt			bi-allelic		del6/-1(A)
**6**	1			wt				wt	
	2			wt				wt	
	3			wt				wt	
	4			wt				wt	
	5			wt				wt	
	6			wt				wt	
**Total**	**29**	**2**	**3**	**24**		**11**	**12**	**6**	

Supplemental Table 6. Genotype and mutations of the rice T2 plants used in this work.

Oryza sativa (Os04g56950)						
**Line**	**Plant**	**Genotype**				**Mutation**
**1**	1	Mut. hom				-1(A)
	2			bi-allelic		-1(A)/-1(C)
**3**	1			bi-allelic		-1(A)/+1(G)
	2	Mut. hom				+A
**4**	1	Mut. hom				+A
	2	Mut. hom				+A
	3	Mut. hom				+A
**5**	1	Mut. hom				+A
	2			bi-allelic		+A/+T
	3	Mut. hom				+A
**6**	1			bi-allelic		+A/+T
	2	Mut. hom				+A
	3	Mut. hom				+T
**8**	1			bi-allelic		+G/+C
**9**	1	Mut. hom				+G
	2	Mut. hom				+C
	3	Mut. hom				+A
**10**	1	Mut. hom				+A
	2	Mut. hom				+A
	3	Mut. hom				-2(CA)
**12**	1			bi-allelic		-1(A)/-2(GA)
**14**	1			bi-allelic		-1(A)/-2(GA)
	2	Mut. hom				-2(GA)
	3			bi-allelic		+A/+T
**16**	1	Mut. hom				"A" > "TT"
**17**	1	Mut. hom				+A
**18**	1	Mut. hom				+A
	3				wt	
**20**	1		het			wt/-2(GA)
	2		het			wt/+A
	3	Mut. hom				+G
**Total**	**31**	**20**	**2**	**8**	**1**	

Supplemental Table 7. Genotype and mutations of the arabidopsis T2 plants from Line 1 used in this work.

Arabidopsis thaliana (At5g55250)																	
		Target 1				Target 2				Target 3							
Line	Plant	Genotype			Mutation	Genotype			Mutation	Genotype			Mutation	Genomic Region Genotype			
	2			wt				wt				wt				wt
	3			wt		Mut. hom			+1(C)			wt		Mut. hom		
	4			wt				wt				wt				wt
	5			wt				wt				wt				wt
	6			wt			het		wt/+1(T)		het		wt/-1(A)		het	
	7			wt		Mut. hom			+1(A)			wt		Mut. hom		
	9			wt		Mut. hom			+1(A)			wt		Mut. hom		
	11			wt				wt		Mut. hom			+1(T)	Mut. hom		
	12			wt				wt				wt				wt
	13			wt				wt				wt				wt
	14			wt				wt				wt				wt
	15			wt				wt			het		wt/+1(T)		het	
	16			wt				wt				wt				wt
	18			wt				wt		Mut. hom			+1(T)	Mut. hom		
	19			wt				wt				wt				wt
	20			wt				wt				wt				wt
1	21			wt				wt				wt				wt
	22			wt				wt				wt				wt
	25			wt				wt			het		wt/+1(T)		het	
	28			wt				wt				wt				wt
	29			wt				wt				wt				wt
	30			wt				wt				wt				wt
	31			wt				wt		Mut. hom			+1(T)	Mut. hom		
	32			wt				wt				wt				wt
	34			wt				wt				wt				wt
	35			wt				wt				wt				wt
	36			wt				wt				wt				wt
	37			wt				wt				wt				wt
	38			wt		Mut. hom			+1(A)			wt		Mut. hom		
	39			wt			het		wt/+1(G)			wt			het	
	42			wt		Mut. hom			+1(A)			wt		Mut. hom		
	43			wt				wt				wt				wt
	44			wt				wt				wt				wt
Total	33	0	0	33		5	2	26		3	3	27		8	4	21

Supplemental Table 8. Genotype and mutations of the arabidopsis T2 plants from Line 2 used in this work.

		***Arabidopsis thaliana* (At5g55250)**														
		**Target 1**				**Target 2**				**Target 3**						
**Line**	**Plant**	**Genotype**			**Mutation**	**Genotype**			**Mutation**	**Genotype**			**Mutation**	**Genomic Region Genotype**		
	1			wt				wt				wt				wt
	2			wt				wt				wt				wt
	3			wt				wt		Mut. hom			+1(T)	Mut. hom		
	4			wt				wt				wt				wt
	5			wt				wt				wt				wt
	6			wt				wt				wt				wt
	7			wt				wt				wt				wt
	8			wt				wt				wt				wt
	9			wt				wt			het		wt/+1(T)		het	
	10			wt				wt				wt				wt
	11			wt				wt				wt				wt
	12			wt			het		wt/-2			wt			het	
	13			wt			het		wt/-2			wt			het	
	14			wt				wt				wt				wt
	15			wt				wt		Mut. hom			+1(T)	Mut. hom		
	16			wt				wt				wt				wt
	17			wt				wt		Mut. hom			+1(T)	Mut. hom		
	18			wt				wt			het		wt/+1(T)		het	
	19			wt				wt				wt				wt
	20			wt				wt		Mut. hom			+1(T)	Mut. hom		
	21			wt				wt				wt				wt
	22			wt			het		wt/+1(C)			wt			het	
	24			wt				wt			het		wt/+1(T)		het	
	25			wt				wt			het		wt/+1(A)		het	
	26			wt				wt				wt				wt
	27			wt				wt				wt				wt
	28			wt				wt		Mut. hom			+1(T)	Mut. hom		
	29			wt				wt				wt				wt
	30			wt				wt				wt				wt
	31			wt				wt				wt				wt
**2**	32			wt				wt				wt				wt
	33			wt				wt		Mut. hom			+1(T)	Mut. hom		
	34			wt				wt				wt				wt
	35			wt				wt				wt				wt
	36			wt				wt		Mut. hom			+1(T)	Mut. hom		
	37			wt				wt		Mut. hom			+1(T)	Mut. hom		
	38			wt				wt				wt				wt
	39			wt				wt				wt				wt
	40			wt				wt		Mut. hom			+1(T)	Mut. hom		
	41			wt				wt				wt				wt
	42			wt				wt				wt				wt
	43			wt				wt		Mut. hom			+1(T)	Mut. hom		
	44			wt				wt				wt				wt
	45			wt			bi-allelic		-1(C)/+1(T)			wt			het	
	46			wt				wt				wt				wt
	47			wt				wt			het		wt/+1(T)		het	
	48			wt				wt				wt				wt
	49			wt				wt		Mut. hom			+1(T)	Mut. hom		
	50			wt				wt				wt				wt
	51			wt				wt			het		wt/+1(T)		het	
	52			wt				wt		Mut. hom			+1(T)	Mut. hom		
	53			wt				wt				wt				wt
	54			wt				wt		Mut. hom			+1(T)	Mut. hom		
	55			wt				wt				wt				wt
	56			wt				wt				wt				wt
	57			wt				wt			het		wt/+1(T)		het	
	58			wt				wt		Mut. hom			+1(T)	Mut. hom		
	59			wt				wt		Mut. hom			+1(T)	Mut. hom		
	60			wt				wt			het		wt/+1(T)		het	
	61			wt				wt		Mut. hom			+1(T)	Mut. hom		
	62			wt				wt				wt				wt
**Total**	**61**	**0**	**0**	**61**		**0**	**4**	**57**		**16**	**8**	**37**		**16**	**12**	**33**

Supplemental Table 9. Genotype and mutations of the arabidopsis T2 plants from Line 3 used in this work.

Arabidopsis thaliana (At5g55250)																
		Target 1				Target 2				Target 3						
Line	Plant	Genotype			Mutation	Genotype			Mutation	Genotype			Mutation	Genomic Region Genotype		
3	1			wt				wt				wt				wt
	2			wt				wt				wt				wt
	3			wt				wt				wt				wt
	4			wt				wt				wt				wt
	5			wt				wt				wt				wt
	6			wt				wt		Mut. hom			+1(T)	Mut. hom		
	7			wt				wt				wt				wt
	8			wt				wt				wt				wt
	9			wt				wt				wt				wt
	10			wt				wt				wt				wt
	11			wt			het		wt/+1(C)			wt			het	
	12			wt				wt				wt				wt
	13			wt				wt				wt				wt
	14			wt				wt				wt				wt
	15			wt				wt				wt				wt
	16			wt				wt				wt				wt
	17			wt				wt			het		wt/+1(T)		het	
	18			wt				wt				wt				wt
	19			wt				wt				wt				wt
	20			wt				wt			het		wt/+1(T)		het	
	21			wt				wt				wt				wt
	22			wt				wt				wt				wt
	23			wt				wt			het		wt/+1(T)		het	
	24			wt				wt				wt				wt
	26			wt				wt			het		wt/+1(T)		het	
	27			wt			het		wt/+1(T)			wt			het	
	28			wt				wt		Mut. hom			+1(T)	Mut. hom		
	29			wt				wt			het		wt/+1(T)		het	
	30			wt				wt				wt				wt
	31			wt				wt				wt				wt
	32			wt				wt				wt				wt
	33			wt				wt				wt				wt
	34			wt				wt				wt				wt
	35			wt		Mut. hom			+1(T)		het		wt/+1(T)		het	
	37			wt				wt			het		wt/+1(T)		het	
	38			wt				wt				wt				wt
	39			wt				wt				wt				wt
	40			wt				wt				wt				wt
	41			wt				wt				wt				wt
	42			wt				wt			het		wt/+1(T)		het	
	43			wt				wt				wt				wt
	44		het		wt/del240		het		wt/del240			wt			het	
	45			wt				wt				wt				wt
	51		het		wt/del240		het		wt/del240			wt			het	
Total	44	0	2	42		1	4	39		2	8	34		2	12	30

Supplemental Table 10. Genotype and mutations of the arabidopsis T2 plants from Line 5 used in this work.

Arabidopsis thaliana (At5g55250)																
		Target 1				Target 2				Target 3						
Line	Plant	Genotype			Mutation	Genotype			Mutation	Genotype			Mutation	Genomic Region Genotype		
5	1			wt			het		wt/+1(A)		het		wt/+1(T)		het	
	2			wt			het		wt/+1(T)		het		wt/+2(TT)		het	
	3			wt				wt				wt				wt
	4			wt				wt				wt				wt
	5			wt				wt		Mut. hom			del4	Mut. hom		
	6			wt				wt				wt				wt
	7			wt				wt			het		wt/+2(TT)		het	
	8			wt				wt			het		wt/+1(T)		het	
	9			wt				wt				wt				wt
	10			wt			bi-allelic		+1(A)/ del193		bi-allelic		+1(T) / del193		het	
	11			wt			het		wt/-1(C)		het		wt/+1(T)		het	
	12			wt				wt			het		wt/del4		het	
	13			wt			bi-allelic		+1(A)/+1(C)		het		wt/+1(T)		het	
	14			wt				wt		Mut. hom			+1(T)	Mut. hom		
	15			wt				wt				wt				wt
	16		het		wt/del3			wt				wt			het	
	17			wt				wt				wt				wt
	18			wt				wt				wt				wt
	19			wt		Mut. hom			del193	Mut. hom			del193	Mut. hom		
	20			wt				wt		Mut. hom			del4	Mut. hom		
	21			wt				wt		Mut. hom			+1(T)	Mut. hom		
	22			wt				wt				wt				wt
	23			wt				wt				wt				wt
	24			wt			bi-allelic		+1(A)/ del193		bi-allelic		+1(T) / del193		het	
	25			wt				wt				wt				wt
	26			wt				wt			het		wt/+2(TT)		het	
	27			wt				wt		Mut. hom				Mut. hom		
	28			wt				wt			het		wt/del4		het	
	29			wt				wt			het		wt/del4		het	
	30			wt				wt			het		wt/del4		het	
	31			wt				wt			het		wt/del4		het	
	32			wt				wt				wt				wt
	33			wt				wt				wt				wt
	34			wt				wt				wt				wt
	35			wt		Mut. hom			del193	Mut. hom			del193	Mut. hom		
	36			wt				wt				wt				wt
	37			wt				wt			het		wt/del4		het	
	38			wt				wt			het		wt/+2(TT)		het	
	39			wt				wt		Mut. hom			+1(T)	Mut. hom		
	40			wt				wt		Mut. hom			+2(TT)	Mut. hom		
	41			wt				wt			het		wt/+2(TT)		het	
	42			wt				wt			het		wt/+1(T)		het	
	43			wt				wt		Mut. hom			+1(T)	Mut. hom		
	44			wt				wt		Mut. hom			+1(T)	Mut. hom		
	45			wt				wt		Mut. hom			+1(T)	Mut. hom		
	46			wt				wt			het		wt/del4		het	
	47			wt				wt		Mut. hom			+1(T)	Mut. hom		
	48			wt				wt				wt				wt
	49			wt		Mut. hom			del193	Mut. hom			del193	Mut. hom		
	50			wt				wt		Mut. hom			del4	Mut. hom		
	51			wt				wt				wt				wt
	52			wt				wt		Mut. hom			del4	Mut. hom		
	53			wt				wt				wt				wt
	54			wt				wt				wt				wt
	55			wt			het		wt/-2(CA)			wt			het	
	56			wt				wt			het		wt/+2(TT)		het	
	57			wt				wt			het		wt/+2(TT)		het	
	58			wt				wt		Mut. hom			+2(TT)	Mut. hom		
	59			wt				wt				wt				wt
	60			wt				wt				wt				wt
	61			wt			het		wt/-2(CA)		het		wt/del4		het	
	62			wt				wt				wt				wt
	63			wt				wt				wt				wt
	64			wt				wt				wt				wt
	65			wt				wt			het		wt/+1(T)		het	
	66			wt				wt				wt				wt
	67			wt				wt		Mut. hom			+1(T)	Mut. hom		
	68			wt				wt			het		wt/del4		het	
	69			wt				wt			het		wt/+2(TT)		het	
	70			wt				wt				wt				wt
	71			wt				wt				wt				wt
	72			wt				wt		Mut. hom			+1(T)	Mut. hom		
	73			wt				wt		Mut. hom			+1(T)	Mut. hom		
	74			wt			het		+1(A)/+1(C)	Mut. hom			+1(T)		het	
	75			wt				wt			het		wt/+2(TT)		het	
	76			wt				wt				wt				wt
**Total**	**76**	**0**	**1**	**75**		**3**	**9**	**64**		**21**	**26**	**29**		**20**	**29**	**27**
